# Supplementary material for: Host Environment Shapes S. aureus Social Behavior as Revealed by Microscopy Pattern Formation and Dynamic Aggregation Analysis
Source: Microorganisms. 2022 Feb 28;10(3):526. doi: 10.3390/microorganisms10030526 (PMC8949161; doi:10.3390/microorganisms10030526)
Supplement: Supplementary file 1 [file microorganisms-10-00526-s001.zip › microorganisms-1580801-supplementary.pdf]

Supplementary Material

Host environment shapes *S. aureus* social behavior by promoting plastic interaction of planktonic and sessile multicellular communities

Natsuko Rivera-Yoshida, Marta Bottagisio, Davide Attanasi, Paolo Savadori, Elena De Vecchi, Alessandro Bidossi, Alessio Franci

Supplementary figures

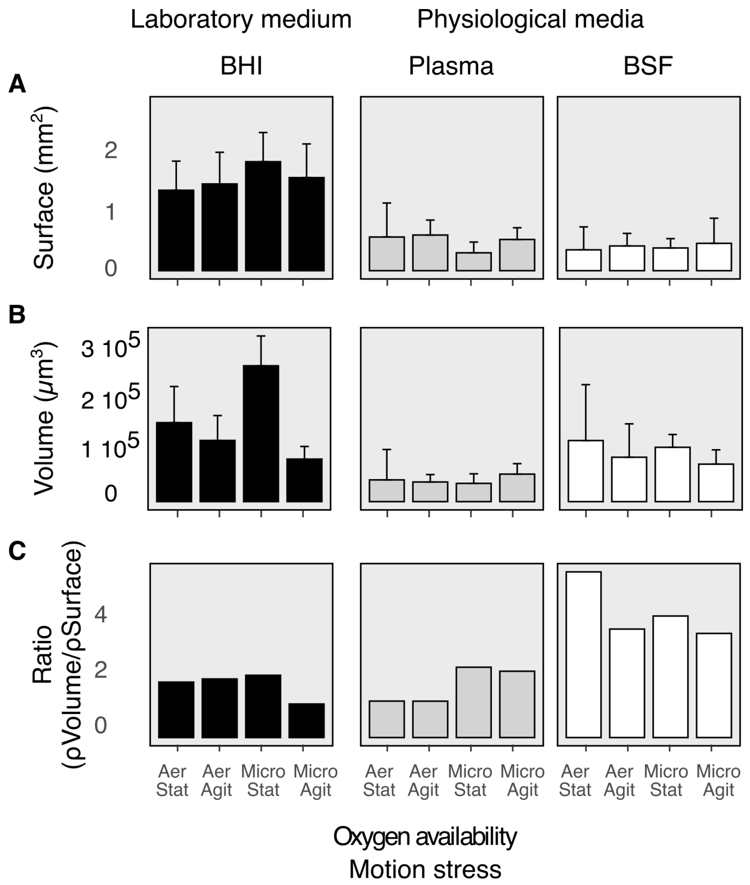

Figure S1: CLSM quantitative analysis of biofilm volume and surface on titanium disks. A) Total surface occupied by sessile biofilm on titanium disk. Horizontal lines represent statistical significance (Wilcoxon, $p < 0.05$). B) Total volume of biofilm biomass. C) Average volume/surface ratio in the different media.

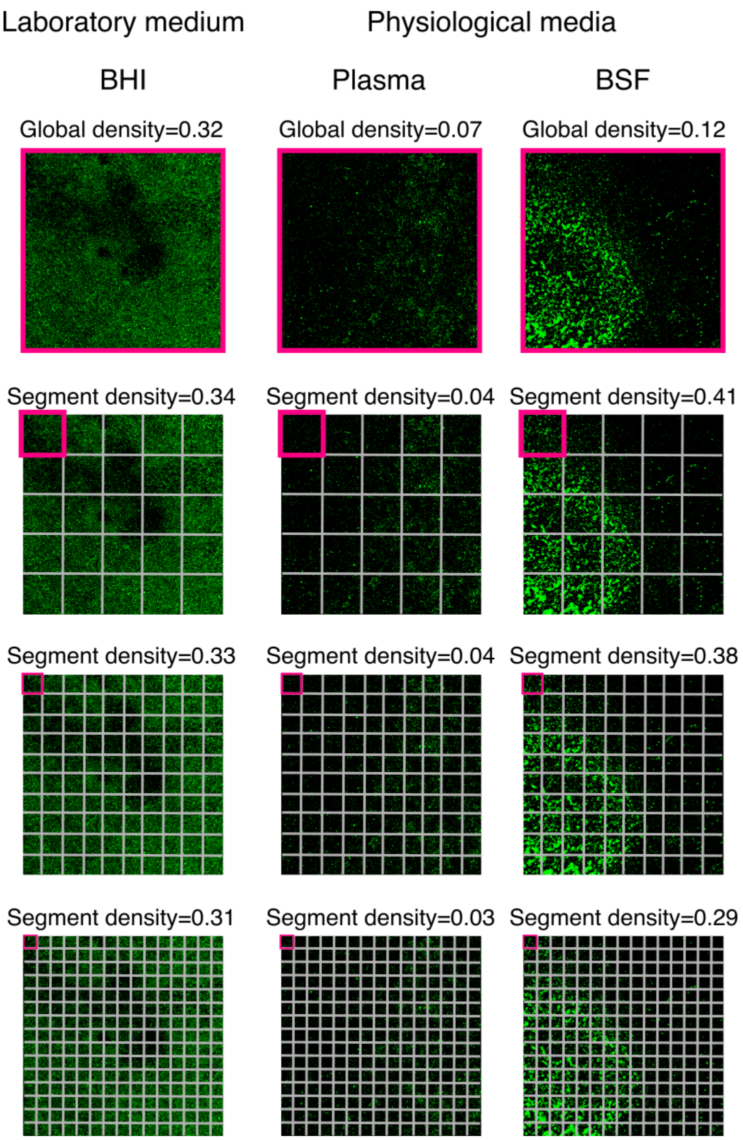

Figure S2: Pattern analysis methodologycal procedure. Confocal microscopy images of sessile biofilm were segmented into grids of 5×5, 6×6 and so on until 15×15 elements and the biofilm density was calculated for each of the resulting grid element.

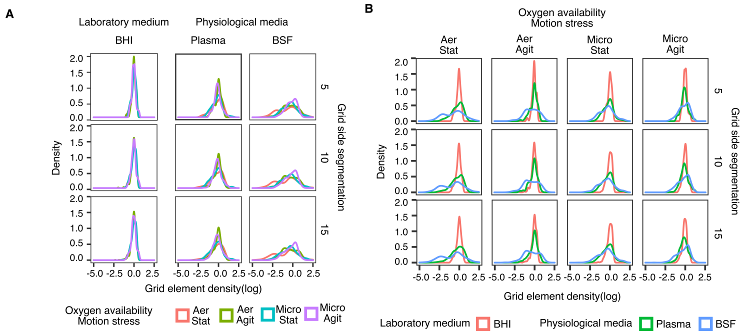

Figure S3: Probability density function of grid elements biofilm density A) across oxygen availability/movement-related stress conditions and B) across different media.

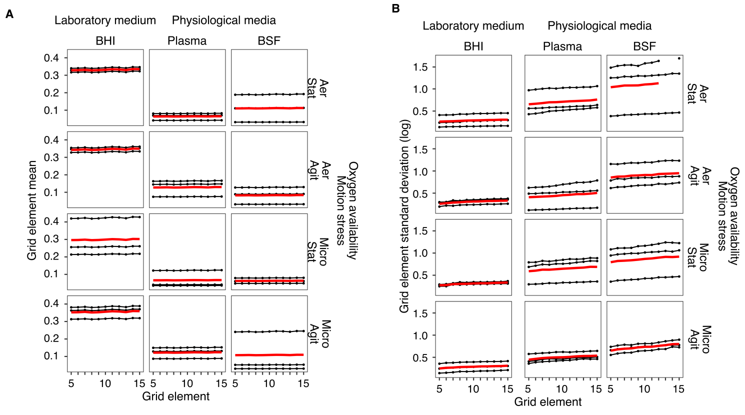

Figure S4: Grid element biofilm density mean (A) and standard deviation (B).

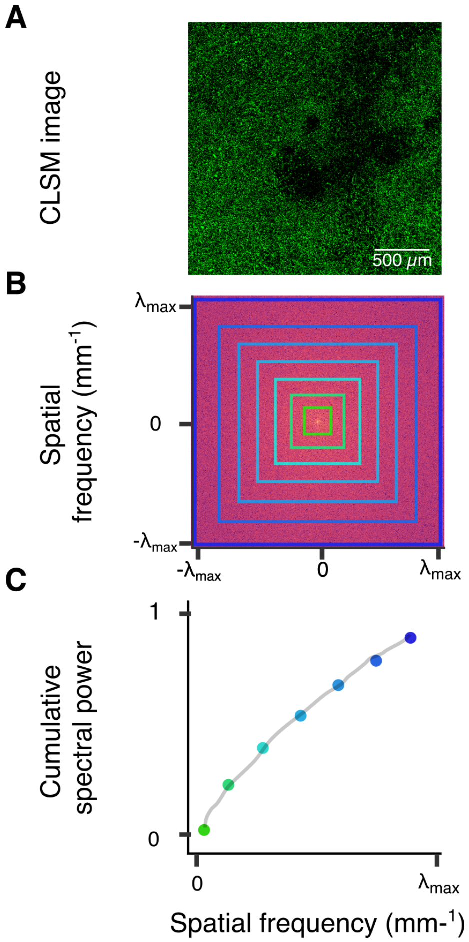

Figure S5: Fourier analysis methodological procedure. A) Confocal microscopy image of sessile biofilm. B) Two-dimensional Fourier power spectrum of the image in A). C) The cumulative spectral power was calculated by integrating the Fourier spectrum matrix over increasingly larger concentric squares.

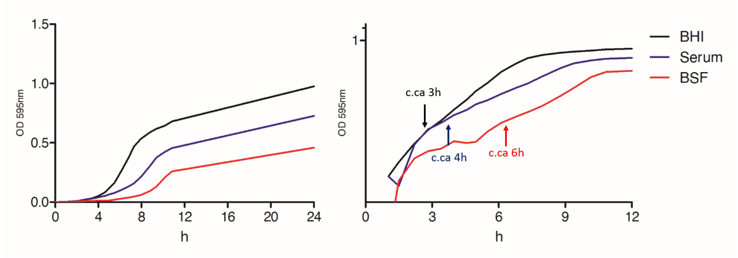

Figure S6: *S. aureus* growth curves in commercial lab medium and physiological fluids. Left panel shows growth displayed in linear scale, growth curve in right panel is displayed in logaritmic scale. Black lines, BHI broth; blue lines, plasma; red lines, BSF.

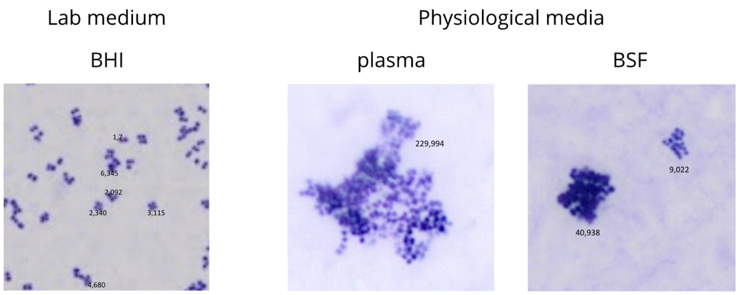

Figure S7: Representative images of *S. aureus* planktonic aggregates after 8 hours of incubation, stained with crystal violet. Left panel displays staphylococcal cells in commercial BHI medium, central and right panels respectively display planktonic aggregates formed in plasma and BSF. In the micrographs, surface of some aggregates is reported in μm^2.

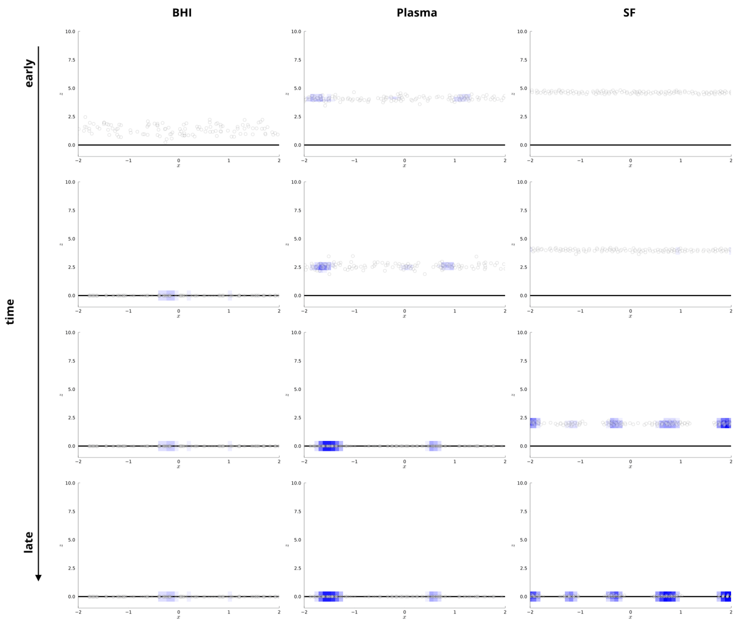

Figure S8: Snapshots of the time evolution of our minimal model for different simulated media (see main text for the used parameters). Each white circle represents a virtual bacterium. The transparent blue heatmap represents the appearance of bacterial aggregates. The darker the blue the larger the aggregates. Aggregates were computed by first measuring bacterial density (computed by counting the number of bacteria in rectangular intervals of horizontal side 0.2 and vertical side 0.5) and then thresholding it to 15 (i.e., only rectangles with at least 15 bacteria were colored). For all rectangles with above-threshold bacterial count, the aggregates size (represented by the blue tone as specified above) was then considered proportional to the number of bacteria falling in the rectangle. The darkest blue tone correspond to 30 or more bacteria in the associated rectangle.

Supplementary movies

Movie S1: Time evolution of our minimal model for BHI parameter set, as specified in the main text. See caption of Figure Figure S8 for a description of the element appearing in each frame.

Movie S2: Time evolution of our minimal model for plasma parameter set, as specified in the main text. See caption of Figure Figure S8 for a description of the element appearing in each frame.

Movie S3: Time evolution of our minimal model for SF parameter set, as specified in the main text. See caption of Figure Figure S8 for a description of the element appearing in each frame.

Supplementary tables

Condition	Medium1	Medium2	p−value Surface	Signif. Surface	p−value Volume	Signif. Volume
Aer_Agit	BHI	Serum	0.004329004	1	0.10000000	0
Aer_Agit	BHI	SF	0.002164502	1	1.00000000	0
Aer_Agit	Serum	SF	0.309523810	0	0.70000000	0
Aer_Stat	BHI	Serum	0.030303030	1	0.01515152	1
Aer_Stat	BHI	SF	0.008658009	1	0.58874459	0
Aer_Stat	Serum	SF	0.428571429	0	0.06493506	0
Micro_Agit	BHI	Serum	0.008658009	1	0.20000000	0
Micro_Agit	BHI	SF	0.008658009	1	1.00000000	0
Micro_Agit	Serum	SF	0.240259740	0	0.40000000	0
Micro_Stat	BHI	Serum	0.002164502	1	0.10000000	0
Micro_Stat	BHI	SF	0.002164502	1	0.10000000	0
Micro_Stat	Serum	SF	0.093073593	0	0.10000000	0

Table S1: Statistical comparison between media for CLSM quantitative analysis of biofilm volume and surface on titanium disks (Wilcoxon, $p < 0.05$).

Medium	Condition1	Condition2	p-value Surface	Signif. Surface	p-value Volume	Signif. Volume
BHI	Aer_Agit	Aer_Stat	0.69913420	0	0.5476190	0
BHI	Micro_Agit	Micro_Stat	0.48484848	0	0.1000000	0
BHI	Aer_Stat	Micro_Stat	0.17965368	0	0.0952381	0
BHI	Micro_Agit	Aer_Agit	0.69913420	0	0.4000000	0
Serum	Aer_Agit	Aer_Stat	0.42857143	0	0.3809524	0
Serum	Micro_Agit	Micro_Stat	0.04112554	1	0.4000000	0
Serum	Aer_Stat	Micro_Stat	0.20021691	0	0.3809524	0
Serum	Micro_Agit	Aer_Agit	0.58874459	0	0.4000000	0
SF	Aer_Agit	Aer_Stat	0.58874459	0	0.5476190	0
SF	Micro_Agit	Micro_Stat	0.69913420	0	0.2000000	0
SF	Aer_Stat	Micro_Stat	0.30952381	0	1.0000000	0
SF	Micro_Agit	Aer_Agit	0.81818182	0	0.7000000	0

Table S2: Statistical comparison between oxygen availability and motion stress conditions for CLSM quantitative analysis of biofilm volume and surface on titanium disks (Wilcoxon, $p < 0.05$).

Medium	Condition	Comp1	Comp2	p value	Wilc p<0.05
* BHI	Aer	Stat	Agit	6.286296E-01	0
BHI	Micro	Stat	Agit	1.387584E-01	0
BHI	Stat	Aer	Micro	6.014389E-01	0
BHI	Agit	Aer	Micro	1.189542E-01	0
SF	Aer	Stat	Agit	2.260145E-07	1
SF	Micro	Stat	Agit	7.855944E-07	1
SF	Stat	Aer	Micro	2.253715E-05	1
SF	Agit	Aer	Micro	3.466501E-03	1
Serum	Aer	Stat	Agit	6.232491E-02	0
Serum	Micro	Stat	Agit	3.365278E-03	1
Serum	Stat	Aer	Micro	5.923340E-02	0
Serum	Agit	Aer	Micro	1.521506E-02	1

ej. * BHI_Aer_Stat vs BHI_Aer_Agit

Condition	Comp1	Comp2	p value	Wilc p<0.05
* Aer Agit	BHI	SF	4.275509E-15	1
Aer Agit	SF	Serum	5.284734E-09	1
Aer Agit	BHI	Serum	5.532236E-02	0
Aer Stat	BHI	SF	6.918644E-32	1
Aer Stat	SF	Serum	1.207683E-15	1
Aer Stat	BHI	Serum	6.948568E-04	1
Micro Agit	BHI	SF	2.903673E-03	1
Micro Agit	SF	Serum	3.476029E-01	0
Micro Agit	BHI	Serum	1.579051E-06	1
Micro Stat	BHI	SF	3.975267E-25	1
Micro Sat	SF	Serum	3.593595E-05	1
Micro Stat	BHI	Serum	1.056696E-11	1

ej. * BHI_Aer_Agit vs SF_Aer_Agit

Table S3: Statistical comparison between oxygen availability and motion stress conditions (left) and between media (right) for the probability density function curves in the pattern analysis (Wilcoxon, $p < 0.05$).

Medium	Condition1	Condition2	P-value	Signif.	Condition	Medium1	Medium2	p-value	Signif.
BHI	Aer_Agit	Aer_Stat	9.997827E-01	0	Aer_Agit	BHI	Serum	1.797617E-04	1
BHI	Micro_Agit	Micro_Stat	9.907970E-01	0	Aer_Agit	BHI	SF	9.359332E-09	1
BHI	Aer_Stat	Micro_Stat	8.286660E-01	0	Aer_Agit	Serum	SF	3.039269E-01	0
BHI	Micro_Agit	Aer_Agit	9.999732E-01	0	Aer_Stat	BHI	Serum	3.801868E-02	1
Serum	Aer_Agit	Aer_Stat	9.399328E-01	0	Aer_Stat	BHI	SF	0.000000E+00	1
Serum	Micro_Agit	Micro_Stat	8.468592E-04	1	Aer_Stat	Serum	SF	1.050271E-13	1
Serum	Micro_Stat	Micro_Stat	2.202317E-02	1	Micro_Agit	BHI	Serum	3.061031E-04	1
Serum	Micro_Agit	Aer_Agit	1.000000E+00	0	Micro_Agit	BHI	SF	2.940568E-08	1
SF	Aer_Agit	Aer_Stat	3.857985E-06	1	Micro_Agit	Serum	SF	3.419247E-01	0
SF	Micro_Agit	Micro_Stat	9.399328E-01	0	Micro_Stat	BHI	Serum	9.091152E-01	0
SF	Micro_Stat	Micro_Stat	6.605033E-04	1	Micro_Stat	BHI	SF	2.773337E-13	1
SF	Micro_Agit	Aer_Agit	9.999732E-01	0	Micro_Stat	Serum	SF	1.597610E-10	1

Table S4: Statistical comparison between oxygen availability and motion stress conditions (left) and between media (right) for the cumulative spectral power functions in the Fourier analysis (Kolmogorov-Smirnov, $p < 0.05$).

Cumulative spectral power

Experiment

Medium1	Medium2	P-value	Signif.
BHI	Serum	1.500726E-02	1
BHI	SF	1.843858E-12	1
Serum	SF	7.855851E-05	1

Model

Medium1	Medium2	P-value	Signif.
BHI	Serum	7.312605E-07	1
BHI	SF	0	1
Serum	SF	2.543466e-05	1

Table S5: Statistical comparison between experimentally measured and simulated cumulative spectral power functions (CSPfs; Kolmogorov-Smirnov, $p < 0.05$). Experimental CSPfs were averaged over oxygen availability and motion stress conditions.
